# Association between regional right ventricular dysfunction and thrombus location in patients with acute pulmonary embolism

**DOI:** 10.1186/2036-7902-6-S1-A17

**Published:** 2014-01-31

**Authors:** Seong Beom Oh, Chan Young Kho, Gi Jun Moon

**Affiliations:** 1 Department of Emergency Medicine, College of Medicine, Dankook University, Cheonan, Republic of Korea

## Background

McConnell's sign is a distinct echocardiographic finding described in patients with acute pulmonary embolism(PE). That is a distinct regional pattern of RV dysfunction, with akinesia of the mid free wall but normal motion at the apex.

## Objective

The aim of this study is to analyze the association between McConnell's sign and thrombus location in patients diagnosed with acute PE using CT scan.

## Patients and methods

From January 2007 through May 2013, 65 patients were diagnosed with acute PE based on CT scan in emergency department. We analyzed retrospectively transthoracic echocardiogram(TTE) of these patients who were performed TTE during admission. 3 patients were excluded because of indeterminable echocardiographic findings due to poor echo window. According to the location of the thrombus based on the CT scan, 62 study subjects were classified into three groups such as main, lobar and segmental pulmonary artery(PA) thrombus group. We investigated the association between McConnell's sign and thrombus location.

## Results

Thrombus was located in main, lobar and segmental PA respectively 43, 13 and 9. 29 of study subjects(46.8%) had McConnell's sign. This finding had a 67.5% sensitivity and a 90.9% specificity for the presence of main PA thrombus, with a positive predictive value of 93.1% and a negative predictive value of 60.6%. The odds ratio of main PA thrombus among the subjects with McConnell's sign was 20.8 times higher than the odds of main PA thrombus among the subjects without McConnell's sign.

## Conclusion

The presence of regional RV dysfunction known as McConnell's sign should raise the level of clinical suspicion for more severe acute PE rather than less severe PE.
